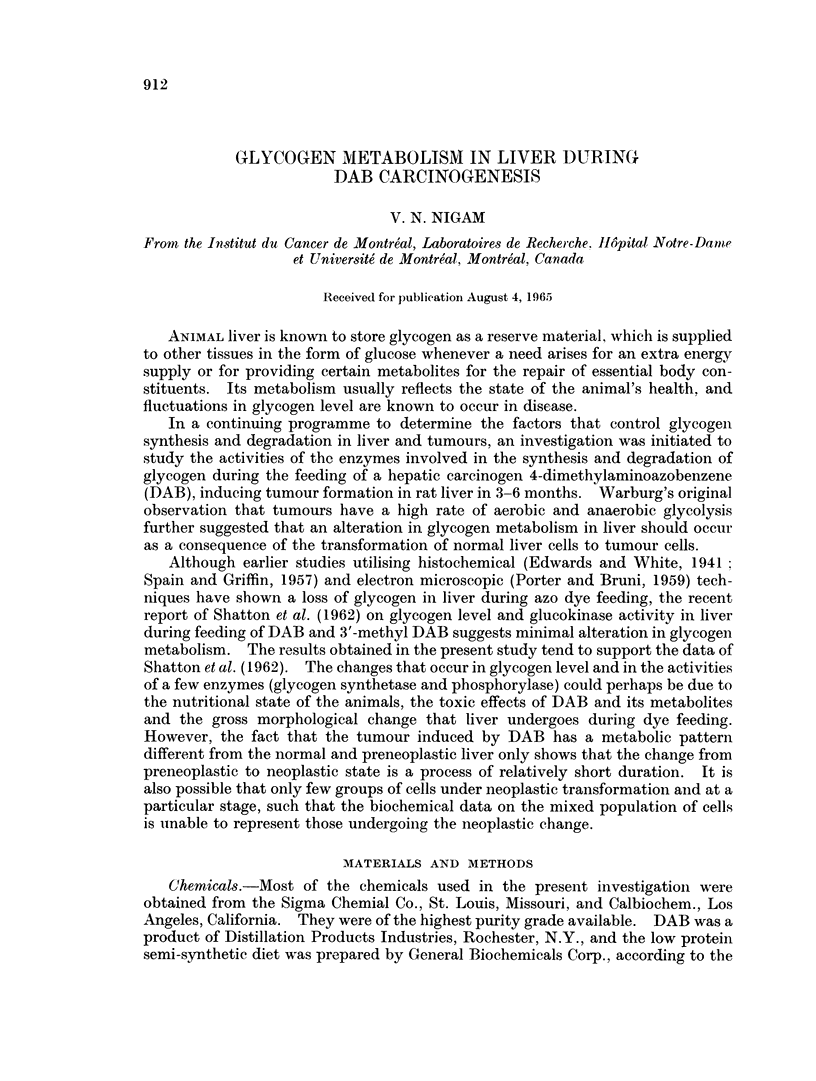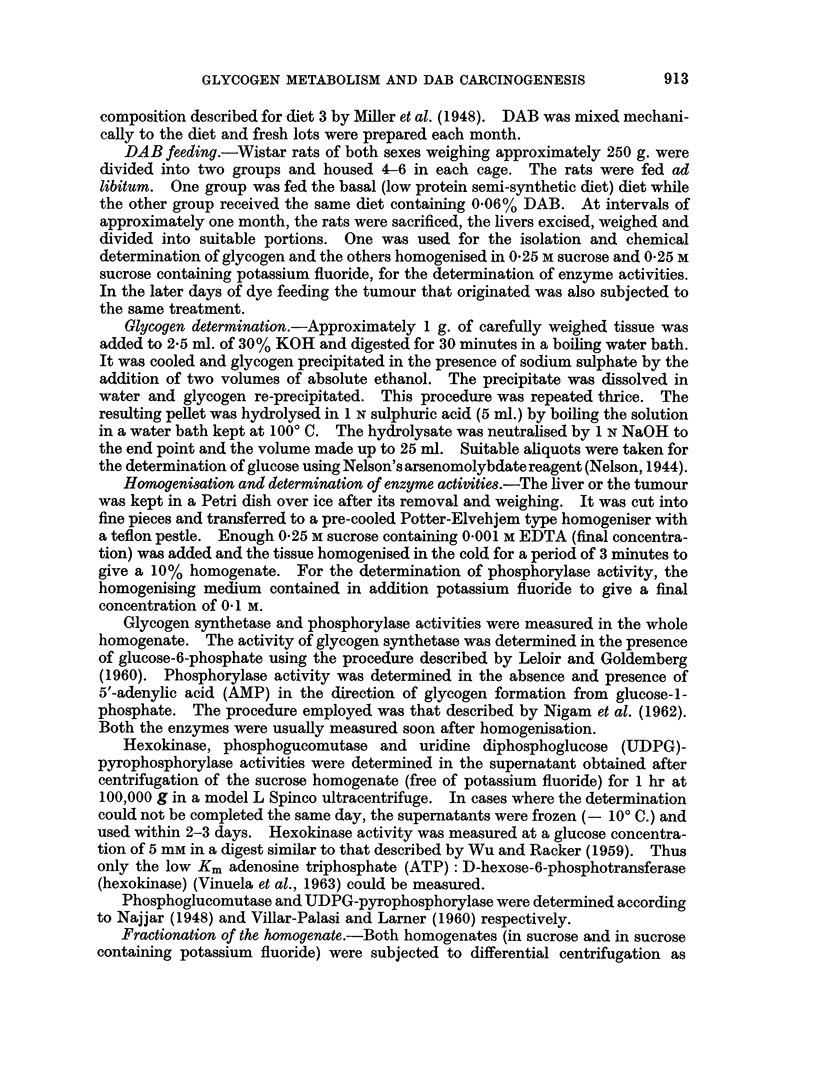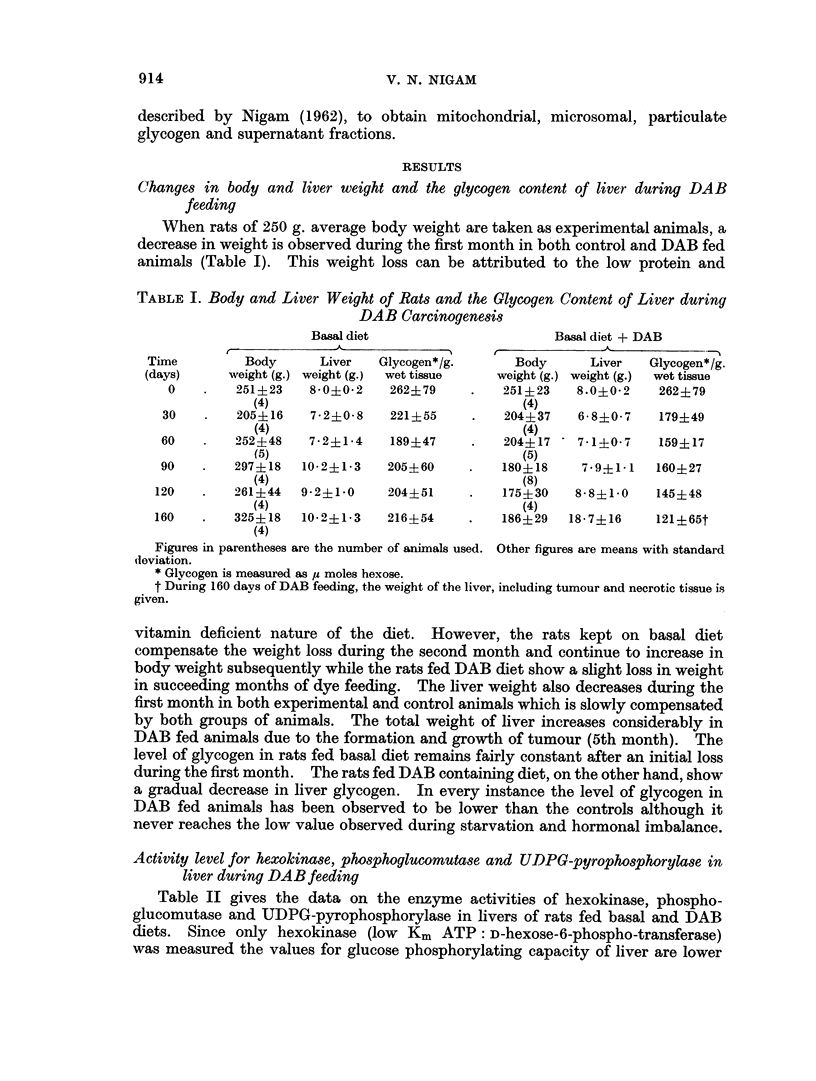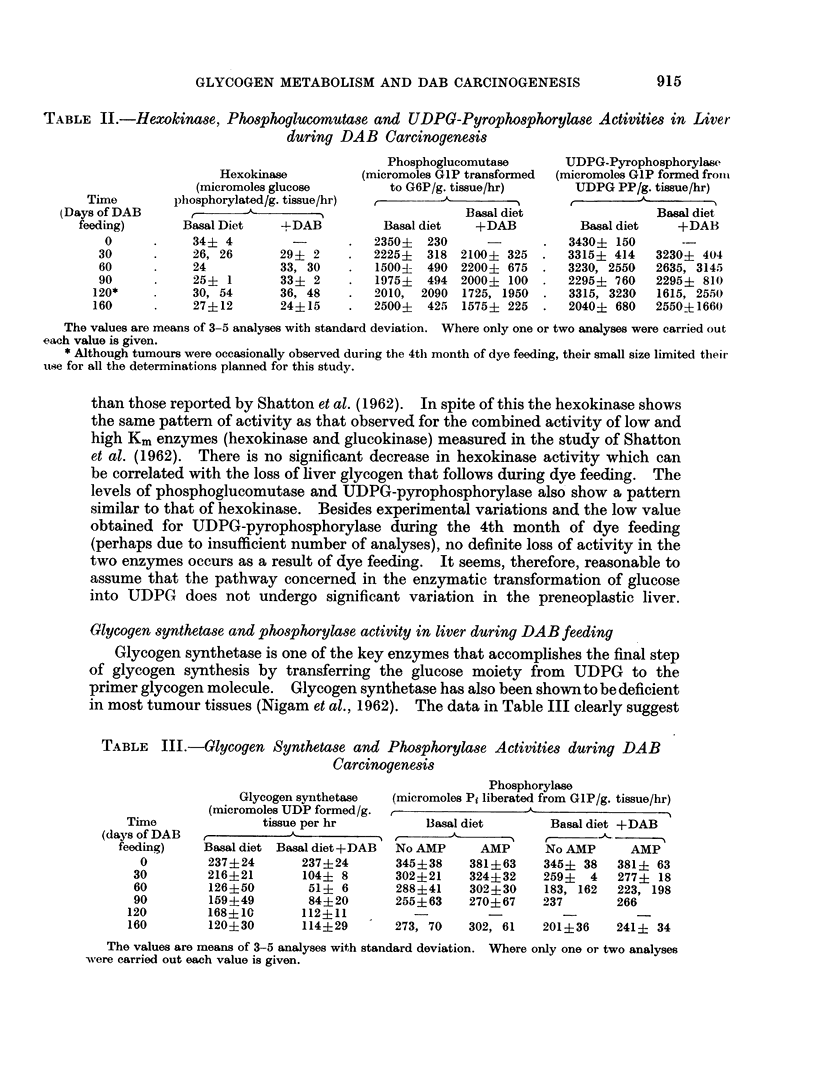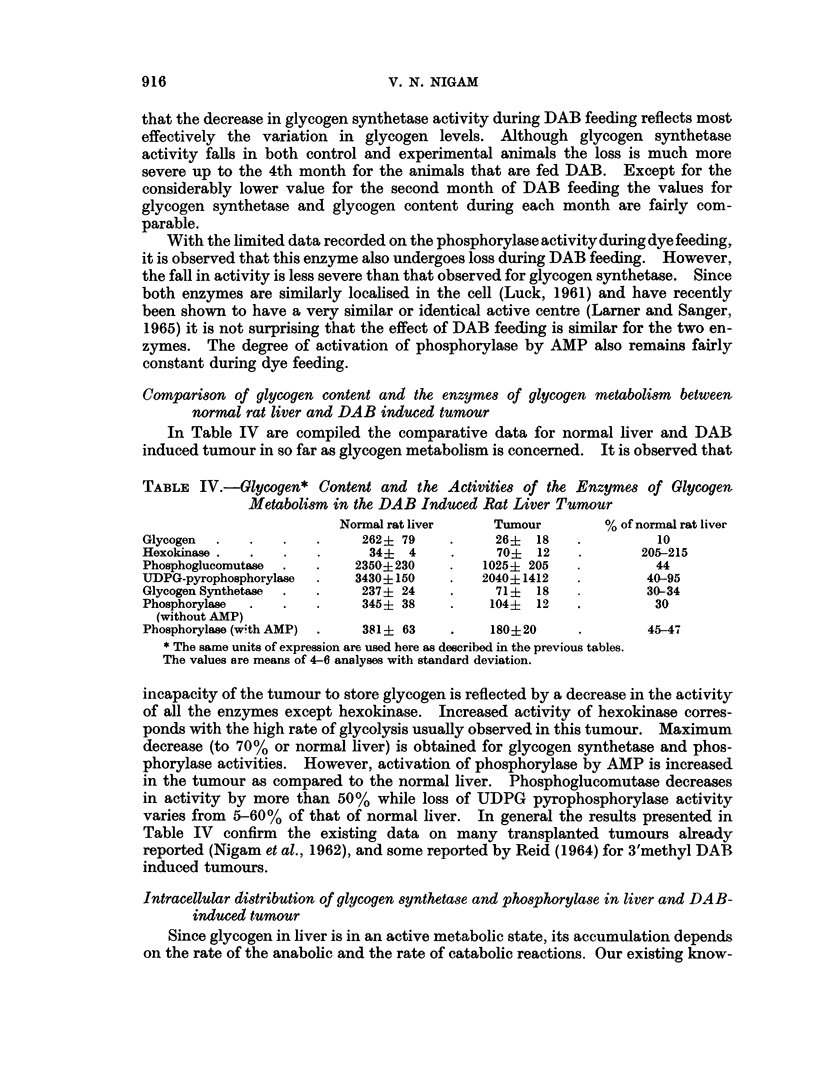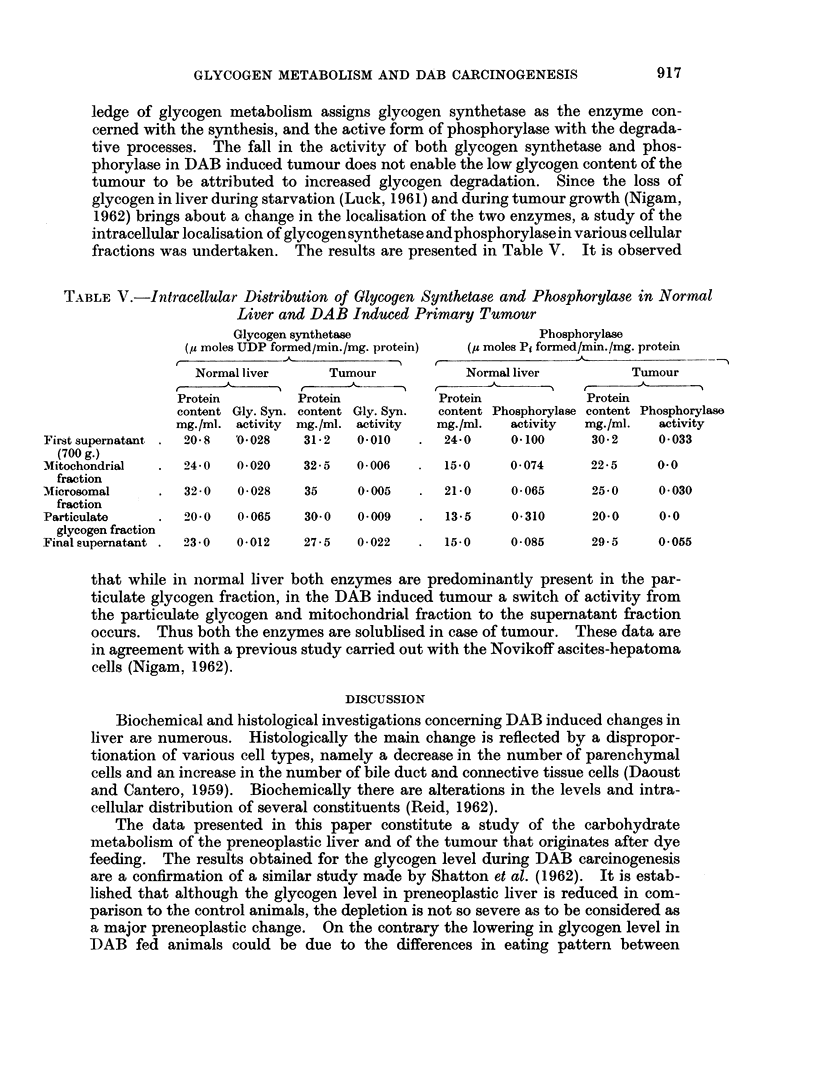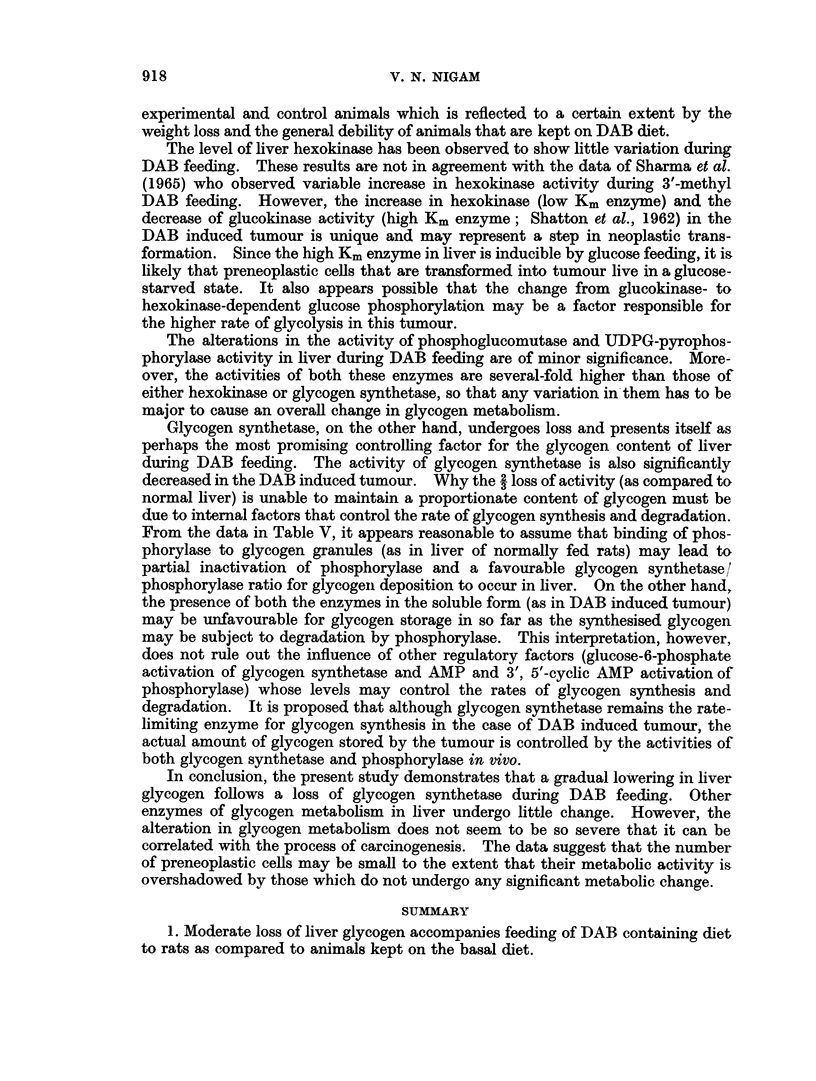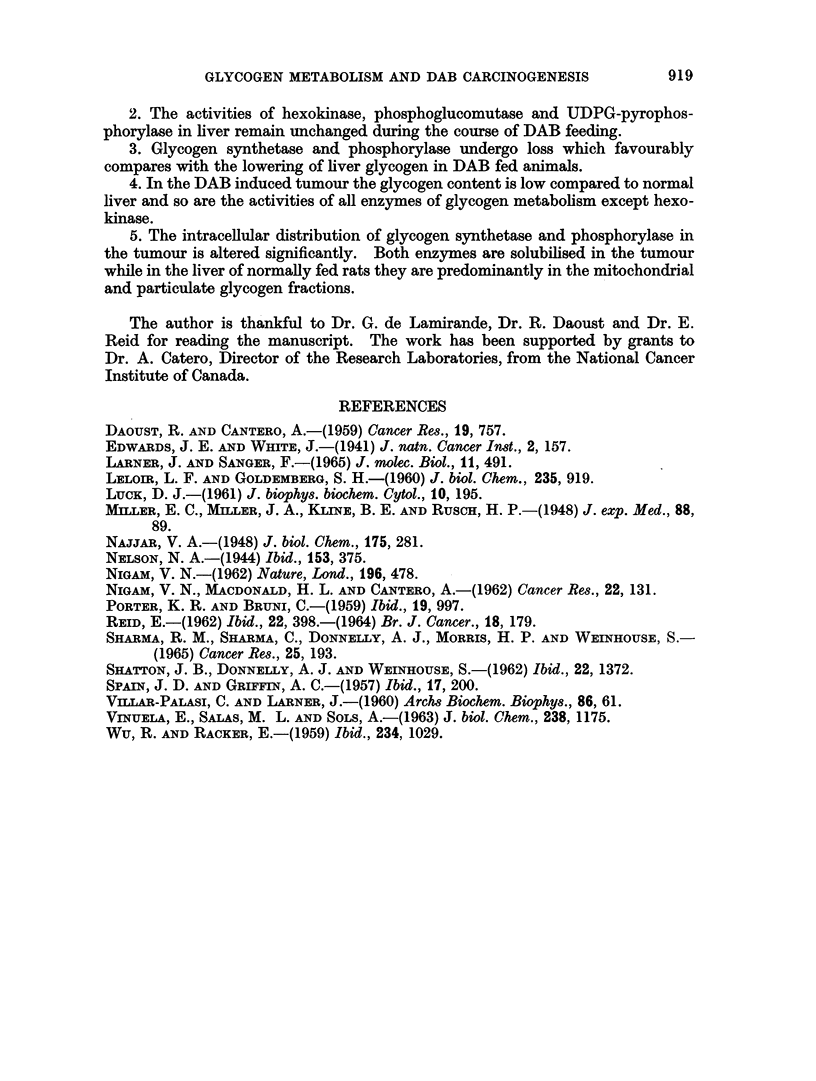# Glycogen metabolism in liver during DAB carcinogenesis.

**DOI:** 10.1038/bjc.1965.106

**Published:** 1965-12

**Authors:** V. N. Nigam


### GLYCOGEN METABOLISM IN LIVER DURING DAB CARCINOGENESIS

V. N. NIGAM

*From the Institut du Cancer de Montréal, Laboratoires de Recherche, Hôpital Notre-Dame et Université de Montréal, Montréal, Canada*

Received for publication August 4, 1965

ANIMAL liver is known to store glycogen as a reserve material, which is supplied to other tissues in the form of glucose whenever a need arises for an extra energy supply or for providing certain metabolites for the repair of essential body constituents. Its metabolism usually reflects the state of the animal's health, and fluctuations in glycogen level are known to occur in disease.

In a continuing programme to determine the factors that control glycogen synthesis and degradation in liver and tumours, an investigation was initiated to study the activities of the enzymes involved in the synthesis and degradation of glycogen during the feeding of a hepatic carcinogen 4-dimethylaminoazobenzene (DAB), inducing tumour formation in rat liver in 3–6 months. Warburg's original observation that tumours have a high rate of aerobic and anaerobic glycolysis further suggested that an alteration in glycogen metabolism in liver should occur as a consequence of the transformation of normal liver cells to tumour cells.

Although earlier studies utilising histochemical (Edwards and White, 1941; Spain and Griffin, 1957) and electron microscopic (Porter and Bruni, 1959) techniques have shown a loss of glycogen in liver during azo dye feeding, the recent report of Shatton *et al.* (1962) on glycogen level and glucokinase activity in liver during feeding of DAB and 3′-methyl DAB suggests minimal alteration in glycogen metabolism. The results obtained in the present study tend to support the data of Shatton *et al.* (1962). The changes that occur in glycogen level and in the activities of a few enzymes (glycogen synthetase and phosphorylase) could perhaps be due to the nutritional state of the animals, the toxic effects of DAB and its metabolites and the gross morphological change that liver undergoes during dye feeding. However, the fact that the tumour induced by DAB has a metabolic pattern different from the normal and preneoplastic liver only shows that the change from preneoplastic to neoplastic state is a process of relatively short duration. It is also possible that only few groups of cells under neoplastic transformation and at a particular stage, such that the biochemical data on the mixed population of cells is unable to represent those undergoing the neoplastic change.

### MATERIALS AND METHODS

*Chemicals.*—Most of the chemicals used in the present investigation were obtained from the Sigma Chemial Co., St. Louis, Missouri, and Calbiochem., Los Angeles, California. They were of the highest purity grade available. DAB was a product of Distillation Products Industries, Rochester, N.Y., and the low protein semi-synthetic diet was prepared by General Biochemicals Corp., according to the composition described for diet 3 by Miller *et al.* (1948). DAB was mixed mechanically to the diet and fresh lots were prepared each month.

*DAB feeding.*—Wistar rats of both sexes weighing approximately 250 g. were divided into two groups and housed 4–6 in each cage. The rats were fed *ad libitum.* One group was fed the basal (low protein semi-synthetic diet) diet while the other group received the same diet containing 0·06% DAB. At intervals of approximately one month, the rats were sacrificed, the livers excised, weighed and divided into suitable portions. One was used for the isolation and chemical determination of glycogen and the others homogenised in 0·25 M sucrose and 0·25 M sucrose containing potassium fluoride, for the determination of enzyme activities. In the later days of dye feeding the tumour that originated was also subjected to the same treatment.

*Glycogen determination.*—Approximately 1 g. of carefully weighed tissue was added to 2·5 ml. of 30% KOH and digested for 30 minutes in a boiling water bath. It was cooled and glycogen precipitated in the presence of sodium sulphate by the addition of two volumes of absolute ethanol. The precipitate was dissolved in water and glycogen re-precipitated. This procedure was repeated thrice. The resulting pellet was hydrolysed in 1 N sulphuric acid (5 ml.) by boiling the solution in a water bath kept at 100° C. The hydrolysate was neutralised by 1 N NaOH to the end point and the volume made up to 25 ml. Suitable aliquots were taken for the determination of glucose using Nelson's arsenomolybdate reagent (Nelson, 1944).

*Homogenisation and determination of enzyme activities.*—The liver or the tumour was kept in a Petri dish over ice after its removal and weighing. It was cut into fine pieces and transferred to a pre-cooled Potter-Elvehjem type homogeniser with a teflon pestle. Enough 0·25 M sucrose containing 0·001 M EDTA (final concentration) was added and the tissue homogenised in the cold for a period of 3 minutes to give a 10% homogenate. For the determination of phosphorylase activity, the homogenising medium contained in addition potassium fluoride to give a final concentration of 0·1 M.

Glycogen synthetase and phosphorylase activities were measured in the whole homogenate. The activity of glycogen synthetase was determined in the presence of glucose-6-phosphate using the procedure described by Leloir and Goldemberg (1960). Phosphorylase activity was determined in the absence and presence of 5′-adenylic acid (AMP) in the direction of glycogen formation from glucose-1-phosphate. The procedure employed was that described by Nigam *et al.* (1962). Both the enzymes were usually measured soon after homogenisation.

Hexokinase, phosphogucomutase and uridine diphosphoglucose (UDPG)-pyrophosphorylase activities were determined in the supernatant obtained after centrifugation of the sucrose homogenate (free of potassium fluoride) for 1 hr at 100,000 *g* in a model L Spinco ultracentrifuge. In cases where the determination could not be completed the same day, the supernatants were frozen (— 10° C.) and used within 2–3 days. Hexokinase activity was measured at a glucose concentration of 5 mM in a digest similar to that described by Wu and Racker (1959). Thus only the low $K_m$ adenosine triphosphate (ATP) : D-hexose-6-phosphotransferase (hexokinase) (Vinuela *et al.*, 1963) could be measured.

Phosphoglucomutase and UDPG-pyrophosphorylase were determined according to Najjar (1948) and Villar-Palasi and Larner (1960) respectively.

*Fractionation of the homogenate.*—Both homogenates (in sucrose and in sucrose containing potassium fluoride) were subjected to differential centrifugation as described by Nigam (1962), to obtain mitochondrial, microsomal, particulate glycogen and supernatant fractions.

### RESULTS

### *Changes in body and liver weight and the glycogen content of liver during DAB feeding*

When rats of 250 g. average body weight are taken as experimental animals, a decrease in weight is observed during the first month in both control and DAB fed animals (Table I). This weight loss can be attributed to the low protein and vitamin deficient nature of the diet. However, the rats kept on basal diet compensate the weight loss during the second month and continue to increase in body weight subsequently while the rats fed DAB diet show a slight loss in weight in succeeding months of dye feeding. The liver weight also decreases during the first month in both experimental and control animals which is slowly compensated by both groups of animals. The total weight of liver increases considerably in DAB fed animals due to the formation and growth of tumour (5th month). The level of glycogen in rats fed basal diet remains fairly constant after an initial loss during the first month. The rats fed DAB containing diet, on the other hand, show a gradual decrease in liver glycogen. In every instance the level of glycogen in DAB fed animals has been observed to be lower than the controls although it never reaches the low value observed during starvation and hormonal imbalance.

TABLE I. *Body and Liver Weight of Rats and the Glycogen Content of Liver during DAB Carcinogenesis*

| | Basal diet | | | Basal diet + DAB | | |
|---|---|---|---|---|---|---|
| Time (days) | Body weight (g.) | Liver weight (g.) | Glycogen*/g. wet tissue | Body weight (g.) | Liver weight (g.) | Glycogen*/g. wet tissue |
| 0 | 251±23 (4) | 8·0±0·2 | 262±79 | 251±23 (4) | 8.0±0·2 | 262±79 |
| 30 | 205±16 (4) | 7·2±0·8 | 221±55 | 204±37 (4) | 6·8±0·7 | 179±49 |
| 60 | 252±48 (5) | 7·2±1·4 | 189±47 | 204±17 (5) | 7·1±0·7 | 159±17 |
| 90 | 297±18 (4) | 10·2±1·3 | 205±60 | 180±18 (8) | 7·9±1·1 | 160±27 |
| 120 | 261±44 (4) | 9·2±1·0 | 204±51 | 175±30 (4) | 8·8±1·0 | 145±48 |
| 160 | 325±18 (4) | 10·2±1·3 | 216±54 | 186±29 | 18·7±16 | 121±65† |

Figures in parentheses are the number of animals used. Other figures are means with standard deviation.

* Glycogen is measured as $\mu$ moles hexose.

† During 160 days of DAB feeding, the weight of the liver, including tumour and necrotic tissue is given.

### *Activity level for hexokinase, phosphoglucomutase and UDPG-pyrophosphorylase in liver during DAB feeding*

Table II gives the data on the enzyme activities of hexokinase, phosphoglucomutase and UDPG-pyrophosphorylase in livers of rats fed basal and DAB diets. Since only hexokinase (low $K_m$ ATP : D-hexose-6-phospho-transferase) was measured the values for glucose phosphorylating capacity of liver are lower

TABLE II.—*Hexokinase, Phosphoglucomutase and UDPG-Pyrophosphorylase Activities in Liver during DAB Carcinogenesis*

| Time (Days of DAB feeding) | Hexokinase (micromoles glucose phosphorylated/g. tissue/hr) | | Phosphoglucomutase (micromoles G1P transformed to G6P/g. tissue/hr) | | UDPG-Pyrophosphorylase (micromoles G1P formed from UDPG PP/g. tissue/hr) | |
|---|---|---|---|---|---|---|
| | Basal Diet | +DAB | Basal diet | Basal diet +DAB | Basal diet | Basal diet +DAB |
| 0 | 34± 4 | — | 2350± 230 | — | 3430± 150 | — |
| 30 | 26, 26 | 29± 2 | 2225± 318 | 2100± 325 | 3315± 414 | 3230± 404 |
| 60 | 24 | 33, 30 | 1500± 490 | 2200± 675 | 3230, 2550 | 2635, 3145 |
| 90 | 25± 1 | 33± 2 | 1975± 494 | 2000± 100 | 2295± 760 | 2295± 810 |
| 120* | 30, 54 | 36, 48 | 2010, 2090 | 1725, 1950 | 3315, 3230 | 1615, 2550 |
| 160 | 27±12 | 24±15 | 2500± 425 | 1575± 225 | 2040± 680 | 2550±1660 |

The values are means of 3–5 analyses with standard deviation. Where only one or two analyses were carried out each value is given.

* Although tumours were occasionally observed during the 4th month of dye feeding, their small size limited their use for all the determinations planned for this study.

than those reported by Shatton *et al.* (1962). In spite of this the hexokinase shows the same pattern of activity as that observed for the combined activity of low and high $K_m$ enzymes (hexokinase and glucokinase) measured in the study of Shatton *et al.* (1962). There is no significant decrease in hexokinase activity which can be correlated with the loss of liver glycogen that follows during dye feeding. The levels of phosphoglucomutase and UDPG-pyrophosphorylase also show a pattern similar to that of hexokinase. Besides experimental variations and the low value obtained for UDPG-pyrophosphorylase during the 4th month of dye feeding (perhaps due to insufficient number of analyses), no definite loss of activity in the two enzymes occurs as a result of dye feeding. It seems, therefore, reasonable to assume that the pathway concerned in the enzymatic transformation of glucose into UDPG does not undergo significant variation in the preneoplastic liver.

*Glycogen synthetase and phosphorylase activity in liver during DAB feeding*

Glycogen synthetase is one of the key enzymes that accomplishes the final step of glycogen synthesis by transferring the glucose moiety from UDPG to the primer glycogen molecule. Glycogen synthetase has also been shown to be deficient in most tumour tissues (Nigam *et al.*, 1962). The data in Table III clearly suggest

TABLE III.—*Glycogen Synthetase and Phosphorylase Activities during DAB Carcinogenesis*

| Time (days of DAB feeding) | Glycogen synthetase (micromoles UDP formed/g. tissue per hr | | Phosphorylase (micromoles $P_i$ liberated from G1P/g. tissue/hr) Basal diet | | Basal diet +DAB | |
|---|---|---|---|---|---|---|
| | Basal diet | Basal diet+DAB | No AMP | AMP | No AMP | AMP |
| 0 | 237±24 | 237±24 | 345±38 | 381±63 | 345± 38 | 381± 63 |
| 30 | 216±21 | 104± 8 | 302±21 | 324±32 | 259± 4 | 277± 18 |
| 60 | 126±50 | 51± 6 | 288±41 | 302±30 | 183, 162 | 223, 198 |
| 90 | 159±49 | 84±20 | 255±63 | 270±67 | 237 | 266 |
| 120 | 168±10 | 112±11 | — | — | — | — |
| 160 | 120±30 | 114±29 | 273, 70 | 302, 61 | 201±36 | 241± 34 |

The values are means of 3–5 analyses with standard deviation. Where only one or two analyses were carried out each value is given.

that the decrease in glycogen synthetase activity during DAB feeding reflects most effectively the variation in glycogen levels. Although glycogen synthetase activity falls in both control and experimental animals the loss is much more severe up to the 4th month for the animals that are fed DAB. Except for the considerably lower value for the second month of DAB feeding the values for glycogen synthetase and glycogen content during each month are fairly comparable.

With the limited data recorded on the phosphorylase activity during dye feeding, it is observed that this enzyme also undergoes loss during DAB feeding. However, the fall in activity is less severe than that observed for glycogen synthetase. Since both enzymes are similarly localised in the cell (Luck, 1961) and have recently been shown to have a very similar or identical active centre (Larner and Sanger, 1965) it is not surprising that the effect of DAB feeding is similar for the two enzymes. The degree of activation of phosphorylase by AMP also remains fairly constant during dye feeding.

*Comparison of glycogen content and the enzymes of glycogen metabolism between normal rat liver and DAB induced tumour*

In Table IV are compiled the comparative data for normal liver and DAB induced tumour in so far as glycogen metabolism is concerned. It is observed that

TABLE IV.—*Glycogen* Content and the Activities of the Enzymes of Glycogen Metabolism in the DAB Induced Rat Liver Tumour*

| | Normal rat liver | Tumour | % of normal rat liver |
|---|---|---|---|
| Glycogen | 262 ± 79 | 26 ± 18 | 10 |
| Hexokinase | 34 ± 4 | 70 ± 12 | 205–215 |
| Phosphoglucomutase | 2350 ± 230 | 1025 ± 205 | 44 |
| UDPG-pyrophosphorylase | 3430 ± 150 | 2040 ± 1412 | 40–95 |
| Glycogen Synthetase | 237 ± 24 | 71 ± 18 | 30–34 |
| Phosphorylase (without AMP) | 345 ± 38 | 104 ± 12 | 30 |
| Phosphorylase (with AMP) | 381 ± 63 | 180 ± 20 | 45–47 |

* The same units of expression are used here as described in the previous tables.
The values are means of 4–6 analyses with standard deviation.

incapacity of the tumour to store glycogen is reflected by a decrease in the activity of all the enzymes except hexokinase. Increased activity of hexokinase corresponds with the high rate of glycolysis usually observed in this tumour. Maximum decrease (to 70% or normal liver) is obtained for glycogen synthetase and phosphorylase activities. However, activation of phosphorylase by AMP is increased in the tumour as compared to the normal liver. Phosphoglucomutase decreases in activity by more than 50% while loss of UDPG pyrophosphorylase activity varies from 5–60% of that of normal liver. In general the results presented in Table IV confirm the existing data on many transplanted tumours already reported (Nigam *et al.*, 1962), and some reported by Reid (1964) for 3′methyl DAB induced tumours.

*Intracellular distribution of glycogen synthetase and phosphorylase in liver and DAB-induced tumour*

Since glycogen in liver is in an active metabolic state, its accumulation depends on the rate of the anabolic and the rate of catabolic reactions. Our existing knowledge of glycogen metabolism assigns glycogen synthetase as the enzyme concerned with the synthesis, and the active form of phosphorylase with the degradative processes. The fall in the activity of both glycogen synthetase and phosphorylase in DAB induced tumour does not enable the low glycogen content of the tumour to be attributed to increased glycogen degradation. Since the loss of glycogen in liver during starvation (Luck, 1961) and during tumour growth (Nigam, 1962) brings about a change in the localisation of the two enzymes, a study of the intracellular localisation of glycogen synthetase and phosphorylase in various cellular fractions was undertaken. The results are presented in Table V. It is observed

TABLE V.—*Intracellular Distribution of Glycogen Synthetase and Phosphorylase in Normal Liver and DAB Induced Primary Tumour*

| | Glycogen synthetase ($\mu$ moles UDP formed/min./mg. protein) | | | | Phosphorylase ($\mu$ moles $P_i$ formed/min./mg. protein) | | | |
|---|---|---|---|---|---|---|---|---|
| | Normal liver | | Tumour | | Normal liver | | Tumour | |
| | Protein content mg./ml. | Gly. Syn. activity | Protein content mg./ml. | Gly. Syn. activity | Protein content mg./ml. | Phosphorylase activity | Protein content mg./ml. | Phosphorylase activity |
| First supernatant (700 g.) | 20·8 | 0·028 | 31·2 | 0·010 | 24·0 | 0·100 | 30·2 | 0·033 |
| Mitochondrial fraction | 24·0 | 0·020 | 32·5 | 0·006 | 15·0 | 0·074 | 22·5 | 0·0 |
| Microsomal fraction | 32·0 | 0·028 | 35 | 0·005 | 21·0 | 0·065 | 25·0 | 0·030 |
| Particulate glycogen fraction | 20·0 | 0·065 | 30·0 | 0·009 | 13·5 | 0·310 | 20·0 | 0·0 |
| Final supernatant | 23·0 | 0·012 | 27·5 | 0·022 | 15·0 | 0·085 | 29·5 | 0·055 |

that while in normal liver both enzymes are predominantly present in the particulate glycogen fraction, in the DAB induced tumour a switch of activity from the particulate glycogen and mitochondrial fraction to the supernatant fraction occurs. Thus both the enzymes are solublised in case of tumour. These data are in agreement with a previous study carried out with the Novikoff ascites-hepatoma cells (Nigam, 1962).

### DISCUSSION

Biochemical and histological investigations concerning DAB induced changes in liver are numerous. Histologically the main change is reflected by a disproportionation of various cell types, namely a decrease in the number of parenchymal cells and an increase in the number of bile duct and connective tissue cells (Daoust and Cantero, 1959). Biochemically there are alterations in the levels and intracellular distribution of several constituents (Reid, 1962).

The data presented in this paper constitute a study of the carbohydrate metabolism of the preneoplastic liver and of the tumour that originates after dye feeding. The results obtained for the glycogen level during DAB carcinogenesis are a confirmation of a similar study made by Shatton *et al.* (1962). It is established that although the glycogen level in preneoplastic liver is reduced in comparison to the control animals, the depletion is not so severe as to be considered as a major preneoplastic change. On the contrary the lowering in glycogen level in DAB fed animals could be due to the differences in eating pattern between experimental and control animals which is reflected to a certain extent by the weight loss and the general debility of animals that are kept on DAB diet.

The level of liver hexokinase has been observed to show little variation during DAB feeding. These results are not in agreement with the data of Sharma *et al.* (1965) who observed variable increase in hexokinase activity during 3′-methyl DAB feeding. However, the increase in hexokinase (low $K_m$ enzyme) and the decrease of glucokinase activity (high $K_m$ enzyme ; Shatton *et al.*, 1962) in the DAB induced tumour is unique and may represent a step in neoplastic transformation. Since the high $K_m$ enzyme in liver is inducible by glucose feeding, it is likely that preneoplastic cells that are transformed into tumour live in a glucose-starved state. It also appears possible that the change from glucokinase- to hexokinase-dependent glucose phosphorylation may be a factor responsible for the higher rate of glycolysis in this tumour.

The alterations in the activity of phosphoglucomutase and UDPG-pyrophosphorylase activity in liver during DAB feeding are of minor significance. Moreover, the activities of both these enzymes are several-fold higher than those of either hexokinase or glycogen synthetase, so that any variation in them has to be major to cause an overall change in glycogen metabolism.

Glycogen synthetase, on the other hand, undergoes loss and presents itself as perhaps the most promising controlling factor for the glycogen content of liver during DAB feeding. The activity of glycogen synthetase is also significantly decreased in the DAB induced tumour. Why the $\frac{2}{3}$ loss of activity (as compared to normal liver) is unable to maintain a proportionate content of glycogen must be due to internal factors that control the rate of glycogen synthesis and degradation. From the data in Table V, it appears reasonable to assume that binding of phosphorylase to glycogen granules (as in liver of normally fed rats) may lead to partial inactivation of phosphorylase and a favourable glycogen synthetase/phosphorylase ratio for glycogen deposition to occur in liver. On the other hand, the presence of both the enzymes in the soluble form (as in DAB induced tumour) may be unfavourable for glycogen storage in so far as the synthesised glycogen may be subject to degradation by phosphorylase. This interpretation, however, does not rule out the influence of other regulatory factors (glucose-6-phosphate activation of glycogen synthetase and AMP and 3′, 5′-cyclic AMP activation of phosphorylase) whose levels may control the rates of glycogen synthesis and degradation. It is proposed that although glycogen synthetase remains the rate-limiting enzyme for glycogen synthesis in the case of DAB induced tumour, the actual amount of glycogen stored by the tumour is controlled by the activities of both glycogen synthetase and phosphorylase *in vivo*.

In conclusion, the present study demonstrates that a gradual lowering in liver glycogen follows a loss of glycogen synthetase during DAB feeding. Other enzymes of glycogen metabolism in liver undergo little change. However, the alteration in glycogen metabolism does not seem to be so severe that it can be correlated with the process of carcinogenesis. The data suggest that the number of preneoplastic cells may be small to the extent that their metabolic activity is overshadowed by those which do not undergo any significant metabolic change.

### SUMMARY

1. Moderate loss of liver glycogen accompanies feeding of DAB containing diet to rats as compared to animals kept on the basal diet.

2. The activities of hexokinase, phosphoglucomutase and UDPG-pyrophosphorylase in liver remain unchanged during the course of DAB feeding.

3. Glycogen synthetase and phosphorylase undergo loss which favourably compares with the lowering of liver glycogen in DAB fed animals.

4. In the DAB induced tumour the glycogen content is low compared to normal liver and so are the activities of all enzymes of glycogen metabolism except hexokinase.

5. The intracellular distribution of glycogen synthetase and phosphorylase in the tumour is altered significantly. Both enzymes are solubilised in the tumour while in the liver of normally fed rats they are predominantly in the mitochondrial and particulate glycogen fractions.

The author is thankful to Dr. G. de Lamirande, Dr. R. Daoust and Dr. E. Reid for reading the manuscript. The work has been supported by grants to Dr. A. Catero, Director of the Research Laboratories, from the National Cancer Institute of Canada.

### REFERENCES

Daoust, R. and Cantero, A.—(1959) *Cancer Res.*, **19**, 757.
Edwards, J. E. and White, J.—(1941) *J. natn. Cancer Inst.*, **2**, 157.
Larner, J. and Sanger, F.—(1965) *J. molec. Biol.*, **11**, 491.
Leloir, L. F. and Goldemberg, S. H.—(1960) *J. biol. Chem.*, **235**, 919.
Luck, D. J.—(1961) *J. biophys. biochem. Cytol.*, **10**, 195.
Miller, E. C., Miller, J. A., Kline, B. E. and Rusch, H. P.—(1948) *J. exp. Med.*, **88**, 89.
Najjar, V. A.—(1948) *J. biol. Chem.*, **175**, 281.
Nelson, N. A.—(1944) *Ibid.*, **153**, 375.
Nigam, V. N.—(1962) *Nature, Lond.*, **196**, 478.
Nigam, V. N., Macdonald, H. L. and Cantero, A.—(1962) *Cancer Res.*, **22**, 131.
Porter, K. R. and Bruni, C.—(1959) *Ibid.*, **19**, 997.
Reid, E.—(1962) *Ibid.*, **22**, 398.—(1964) *Br. J. Cancer.*, **18**, 179.
Sharma, R. M., Sharma, C., Donnelly, A. J., Morris, H. P. and Weinhouse, S.—(1965) *Cancer Res.*, **25**, 193.
Shatton, J. B., Donnelly, A. J. and Weinhouse, S.—(1962) *Ibid.*, **22**, 1372.
Spain, J. D. and Griffin, A. C.—(1957) *Ibid.*, **17**, 200.
Villar-Palasi, C. and Larner, J.—(1960) *Archs Biochem. Biophys.*, **86**, 61.
Vinuela, E., Salas, M. L. and Sols, A.—(1963) J. *biol. Chem.*, **238**, 1175.
Wu, R. and Racker, E.—(1959) *Ibid.*, **234**, 1029.